# Künstliche Intelligenz auf dem Prüfstand: Anforderungen, Qualitätskriterien und Prüfwerkzeuge für medizinische Anwendungen

**DOI:** 10.1007/s00103-025-04101-w

**Published:** 2025-07-14

**Authors:** Jackie Ma, Eva Weicken, Frederik Pahde, Katharina Weitz, Sebastian Lapuschkin, Wojciech Samek, Thomas Wiegand

**Affiliations:** 1 Abteilung für Künstliche Intelligenz, Fraunhofer Heinrich-Hertz-Institut, HHI, Einsteinufer 37, 10587 Berlin, Deutschland; 2 Fakultät IV – Elektrotechnik und Informatik, Technische Universität Berlin, Berlin, Deutschland; 3 Centre of eXplainable Artificial Intelligence, Technical University Dublin, Dublin, Irland; 4 Berlin Institute for the Foundations of Learning and Data (BIFOLD), Berlin, Deutschland

**Keywords:** Qualitätskriterien, Prüfkriterien, Prüfwerkzeuge, Erklärbarkeit, Standardisierung, Quality criteria, Testing criteria, Testing tools, Explainability, Standardization

## Abstract

Der Einsatz von künstlicher Intelligenz (KI) in der Medizin bietet großes Potenzial zur Steigerung von Qualität und Effizienz. Gleichzeitig sind mit ihrer Anwendung Risiken verbunden. Um potenzielle Schäden zu vermeiden, entwickeln Expert:innen aus Forschung und Politik Anforderungskriterien, Prüfwerkzeuge und Richtlinien zur Identifikation und Kontrolle dieser Risiken. Im Mittelpunkt stehen dabei regulatorische und klinische Anforderungen an KI sowie Anforderungen aus Sicht der Nutzer:innen. Diese unterschiedlichen Perspektiven sind bei der Entwicklung von Qualitätskriterien und Prüfwerkzeugen zu berücksichtigen.

In diesem Artikel werden zunächst die Anforderungen an künstliche Intelligenz (KI) in der Medizin erläutert. Anschließend wird dargestellt, wie sich aus diesen Anforderungskriterien konkrete Qualitätskriterien und Prüfwerkzeuge ableiten lassen. Am Beispiel der „Erklärbarkeit“ wird ein zentrales Qualitätskriterium vertiefend behandelt. Zudem wird ein Prüfschema für den Einsatz von KI in der Medizin vorgestellt. Abschließend erfolgt ein Ausblick auf die Standardisierung von Qualitätskriterien.

Die Entwicklung von KI, insbesondere im medizinischen Bereich, schreitet rasant voran. Geeignete Prüfverfahren können dazu beitragen, Risiken zu minimieren und das Vertrauen in die Technologie zu stärken. Für eine erfolgreiche Umsetzung von Prüfverfahren in der Praxis sind jedoch kontinuierliche Forschungsanstrengungen erforderlich.

## Abstract (en)

The use of artificial intelligence (AI) in medicine has great potential to improve quality and efficiency. At the same time, its use is associated with risks. In order to prevent harm, experts from research and politics are developing requirements, testing tools, and guidelines to identify and control these risks.

This article first describes the requirements for AI in medicine. It then follows with a presentation of how concrete quality criteria and testing tools can be derived from those requirements. A deeper demonstration of “explainability” as a key criterion is presented as an example. A testing framework for the use of AI in medicine is also presented. Finally, the article provides an outlook on standardization of quality criteria.

The development of AI, especially in medicine, is progressing quickly. Suitable testing procedures can help to minimize risks and strengthen trust in the technology. However, for the successful implementation of such testing procedures, continuous research efforts are necessary.

## Hintergrund

Künstliche Intelligenz (KI) hat den Status einer reinen Vision längst hinter sich gelassen und ist in vielen Anwendungen bereits allgegenwärtig. Die Medizin stellt ein besonders sensibles Anwendungsfeld für KI dar, da Fehlentscheidungen hier direkte und unmittelbare Auswirkungen auf Leben und Gesundheit von Patient:innen haben können – umso wichtiger sind klare Prüf- und Qualitätskriterien. Damit KI-Anwendungen in der Praxis auch genutzt werden, ist darüber hinaus sicherzustellen, dass Ärzt:innen und Patient:innen ein angemessenes Vertrauen in diese Systeme haben. Ob wir einer neuen Technologie wie der KI vertrauen, hängt wesentlich davon ab, was wir über sie wissen und wie gut wir sie verstehen. Wissen – oder auch Unwissen – bestimmt maßgeblich unsere Fähigkeit, potenzielle Auswirkungen und Risiken ihres Einsatzes einzuschätzen. Dafür werden zugängliche Methoden und Verfahren benötigt, die eine Beurteilung des KI-Modells erlauben. In diesem Zusammenhang sind sowohl Vertrauen als auch Reliabilität zentrale Aspekte: Vertrauen beschreibt das subjektive Sicherheitsempfinden der Nutzenden, während Reliabilität die objektive Leistungsfähigkeit von KI-Systemen bewertet. Dafür sind Prüfkriterien erforderlich, die feststellen, ob eine KI-Anwendung eine Aufgabe anforderungsgerecht erfüllt. Ergänzend dazu braucht es geeignete Prüfwerkzeuge, mit denen die Prüfkriterien nachgewiesen bzw. gemessen werden können. Doch wie können solche Prüfkriterien und Prüfwerkzeuge konkret aussehen?

Der Artikel behandelt Anforderungen an KI in der Medizin und zeigt, wie daraus Qualitätskriterien und Prüfverfahren abgeleitet werden können. Am Beispiel der Erklärbarkeit wird ein zentrales Kriterium vertiefend dargestellt. Zudem wird ein Prüfschema vorgestellt und ein Ausblick auf die Standardisierung von Prüfverfahren gegeben.

## Anforderungen an KI im medizinischen Kontext

Bei der Festlegung von Anforderungskriterien für den Einsatz von KI in der Medizin müssen verschiedene Perspektiven berücksichtigt werden. Dazu gehören regulatorische und klinische Anforderungen und auch Anforderungen aus der Perspektive der Nutzer:innen. Diese Anforderungen können sich überschneiden und aus einer gemeinschaftlichen Perspektive entwickelt werden. Ein besonderes Augenmerk liegt bei der Erstellung solcher Anforderungsprofile auf der Harmonisierung der unterschiedlichen Anforderungen. Ein Widerspruch der unterschiedlichen Anforderungen ist grundsätzlich zu vermeiden.

### Regulatorische Anforderungen

Es existieren bereits regulatorische Anforderungen und hohe Standards, die die Einführung von Medizinprodukten regeln. Gesetze, die für eine Zulassung von Medizinprodukten zu berücksichtigen sind, sind beispielsweise das Medizinprodukterecht-Durchführungsgesetz (MPDG; [1]) und die europäische Medizinprodukteverordnung (EU) 2017/745 (auch Medical Device Regulation, MDR; [2]). Auf dieser gesetzlichen Grundlage folgen beispielsweise die Entwicklung und Zulassung von „klassischer“ Software und Medizinprodukten oder von Medikamenten einem standardisierten, fest etablierten Prozess (Validierung, Testung, klinische Studien und Zertifizierung), um sicherzustellen, dass der Nutzen das Risiko überwiegt und kein Schaden für den Menschen entsteht. Der gleiche Anspruch gilt auch für KI – jedoch lässt sich dieser Prozess nicht eins zu eins auf KI als Medizinprodukt oder „Software as a Medical Device“ (SaMD) übertragen.

Ein wesentlicher Unterschied zwischen „traditionellen“ Medizinprodukten und KI-Anwendungen liegt in ihrer Funktionsweise: Während klassische Medizinprodukte typischerweise auf fest definierten Regeln und Algorithmen basieren, nutzen KI-Systeme häufig maschinelle Lernverfahren, um aus großen Datenmengen Muster zu erkennen und Entscheidungen abzuleiten. Aufgrund der Komplexität dieser Lernverfahren und der daraus resultierenden KI-Modelle ist die innere Entscheidungslogik – im Gegensatz zu klassischen Medizinprodukten – häufig nicht direkt nachvollziehbar. Man spricht hier von „Blackbox“-Modellen. Bei „traditionellen“ Medizinprodukten und Softwareanwendungen werden Risiken in der Regel vorab quantifiziert und durch standardisierte Tests abgesichert, die auf fest definierten, reproduzierbaren Prozessen basieren. Beispielsweise arbeitet ein Magnetresonanztomographie(MRT)-Gerät auf Basis fest definierter physikalischer Prinzipien und standardisierter Bildgebungsprotokolle, die durch regelmäßige Tests reproduzierbare Ergebnisse liefern und deren Regulierung fest etabliert ist. Bei KI-Modellen ist es aufgrund ihres Blackbox-Charakters deutlich schwieriger, die korrekte Funktionsweise zu testen und zu validieren. Hinzu kommt, dass adaptive KI-Systeme sich im laufenden Betrieb unter neuen Daten verändern, im Gegensatz zu „traditionellen“ Medizinprodukten, die meist statisch sind. Zudem hängt ihre Leistungsfähigkeit stark von der Qualität der zugrunde liegenden Daten ab, wodurch sie anfällig für Verzerrungen (Bias) und damit verbundene fehlerhafte Ergebnisse sowie unvorhersehbare Risiken werden kann. Aufgrund dieser Unterschiede sind für KI-gestützte Anwendungen neue Regulierungsmechanismen und Prüfverfahren notwendig.

Die Entwicklung KI-spezifischer Standards und Regularien, die sowohl den spezifischen Anforderungen von KI-Systemen gerecht werden als auch kulturelle und länderspezifische Unterschiede berücksichtigen, ist daher unerlässlich. Dabei ist ein multidisziplinärer Ansatz von Vorteil, da Expert:innen aus unterschiedlichen Fachbereichen – etwa Datenschutzrecht, Standardisierung, Regulierung, Ethik (z. B. Mitglieder des Deutschen Ethikrats), Medizin und Versorgung sowie Softwareentwicklung – jeweils ihre spezifischen Erfahrungen und Anforderungen aus der jeweiligen Anwendungsperspektive einbringen. Dies kann einerseits dazu beitragen, eine erhöhte Sicherheit zu gewährleisten und eine starke Regulation zu fördern, andererseits steigt auch das Risiko einer Überregulierung und dadurch einer möglichen Verlangsamung des Innovationsfortschritts und der Produkteinführung.

Im April 2021 wurde von der Europäischen Kommission erstmals ein Gesetzesentwurf zur Schaffung eines regulativen Rahmens für KI innerhalb der Europäischen Union vorgestellt. Der EU Artificial Intelligence Act (EU AI Act [3]), der am 13.03.2024 verabschiedet wurde, ist das erste umfassende KI-Gesetz weltweit, welches als konkretes legislatives Instrument einen klaren rechtlichen Rahmen für die Entwicklung und den Einsatz von KI in der EU schafft. Bereits im EU AI Act werden verschiedene Qualitätskriterien wie „Genauigkeit“ und „Robustheit“ verankert. Durch das Gesetz sollen Risiken, die mit dem Einsatz von KI verbunden sind, minimiert werden. Hierbei werden die KI-Systeme risikobasiert in die Kategorien „unannehmbares“, „hohes“ und „begrenztes“ Risiko eingeteilt, wobei jeweils unterschiedliche regulatorische Anforderungen gelten. Die praktische Umsetzung und die Implementierung dieses Gesetzes bringen aktuell noch einige Herausforderungen mit sich.

### Klinische Anforderungen an eine KI

Zusätzlich zu den regulatorischen Anforderungen ist es entscheidend, dass eine KI vor ihrem Einsatz in einem komplexen klinischen Setting ausreichend evaluiert wird. Dadurch soll, ähnlich wie bei anderen Medizinprodukten, insbesondere auch das Nutzen-Risiko-Verhältnis nachgewiesen werden. KI sollte nicht nur sicher sein, sondern auch den beabsichtigten Nutzen erfüllen und effektiv eingesetzt werden. Es gibt einige Beispiele, bei denen KI-Systeme zwar unter „Laborbedingungen“ gut funktionieren, jedoch in der Praxis oder wenn sie in einem anderen Setting (z. B. andere Klinik oder anderes Land) implementiert werden, fehlerhaft sind (z. B. [4]) und ggf. sogar Schaden anrichten können. Eine klinische Evaluation von KI-Systemen, einschließlich der technischen Leistungsbewertung und der klinischen Validierung im Rahmen klinischer KI-Studien, ist daher unerlässlich.

**Abb. 1:**
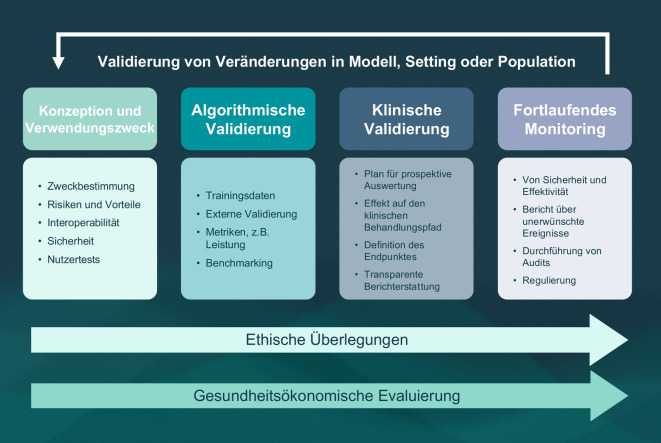
Framework „Clinical Evaluation of AI for Health“ mit Darstellung der 4 Evaluationsphasen und übergreifender ethischer Überlegungen und gesundheitsökonomischer Evaluation. (Ins Deutsche übersetzt, ergänzt und farblich modifiziert aus [5]. Mit freundlicher Genehmigung der ITU)

Ein Beispiel hierfür ist das von der internationalen Expert:innengruppe „Clinical Evaluation of AI for Health“ entwickelte „Framework zur klinischen Evaluierung von KI in Gesundheitsanwendungen“ (FG-AI4H DEL7.4, 2023; [5]). Die Arbeitsgruppe ist Teil der „Focus Group on Artificial Intelligence for Health“ (FG-AI4H; [6]), die von der Internationalen Fernmeldeunion (ITU) und der Weltgesundheitsorganisation (WHO) ins Leben gerufen wurde. Ihr Framework gliedert den klinischen Evaluierungsprozess in 4 Phasen – mit jeweils relevanten Anforderungen – und beinhaltet darüber hinaus wesentliche und phasenübergreifende ethische und gesundheitsökonomische Evaluationsaspekte (Abb. 1):

4 Phasen der klinischen Evaluation:

- *Evaluation von Konzeption und Verwendungszweck: *Diese Phase beinhaltet beispielsweise die Evaluierung des Nutzens und des Verwendungszwecks des KI-Modells. Dazu gehören die Beschreibung des zugrunde liegenden Problems, die Begründung für den Einsatz von KI zur Lösung dieses Problems sowie die Identifikation möglicher Risiken.
- *Algorithmische Validierung:* In-silico-Bewertung der Eignung des KI-Modells, d. h. der technischen Aspekte und Daten, die zur Entwicklung des Modells verwendet wurden. Dazu gehören z. B. Metriken und das Benchmarking im Vergleich zu aktuellen Goldstandards.
- *Klinische Validierung: *Evaluierung des Modells entlang des klinischen Behandlungspfades durch KI-spezifische klinische Studien. Dies umfasst unter anderem die Beschreibung potenzieller Bias, die Charakterisierung des klinischen Settings sowie eine transparente Dokumentation der Studienergebnisse.
- *Fortlaufendes Monitoring: *Kontinuierliche Evaluierung der Leistung und der Auswirkungen des KI-Systems nach Implementierung im klinischen Setting. Beispiele sind der Einsatz eines Entscheidungsunterstützungssystems zur Erkennung von Brustkrebs in der Radiologie, die Durchführung von Audits zur Identifikation unerwarteter Fehler sowie die laufende Prüfung regulatorischer Anforderungen.

Phasenübergreifende Evaluationsaspekte:

- *Ethische Überlegungen*: Diese umfassen grundlegende ethische Anforderungen an die Konzeption, Entwicklung und Implementierung medizinischer KI-Systeme, wie z. B. den Schutz der menschlichen Autonomie, die Förderung von Wohlergehen und Sicherheit, die Wahrung des öffentlichen Interesses sowie die Sicherstellung von Inklusivität und Chancengleichheit (Details siehe [7]).
- *Gesundheitsökonomische Evaluierung:* Darunter versteht man eine vergleichende Analyse mehrerer (gesundheits-)ökonomischer Interventionen hinsichtlich ihrer Kosten und Auswirkungen, bei Umsetzung in einem spezifischen Anwendungskontext. Dazu zählen u. a. die Bewertung der relativen Wirksamkeit der jeweiligen Intervention sowie die Analyse der damit verbundenen Kosten bzw. potenziellen Kosteneinsparungen.

Die regulatorischen und fachlichen (in diesem Fall klinischen) Anforderungen dienen der Sicherheit und Nutzbarkeit von KI-Systemen in der Medizin. Sie stellen sicher, dass ein KI-Modell, sofern diese Anforderungen erfüllt sind, sicher eingesetzt werden kann und einen Mehrwert für die Nutzer:innen bietet. Gleichzeitig schaffen insbesondere die regulatorischen Anforderungen einen rechtlichen Rahmen zur Klärung von Haftungsfragen. Im Falle von KI in der Medizin sind diese neuen Ansätze nötig, denn sowohl die klare Zuordnung hinsichtlich Haftungsfragen (Fehlfunktion vs. Fehlnutzung) als auch die garantierte Sicherheit der KI-Modelle (Prüfbarkeit einer eindeutigen Funktionalität) sind noch nicht vollständig gegenwärtig wie in anderen Bereichen. Das betrifft insbesondere selbstlernende KI-Systeme in der Medizin.

### Anforderungen der Nutzer:innen

Neben den regulatorischen und fachlichen Anforderungen gibt es oftmals weitere Aspekte, die für die Qualitätsbeurteilung von KI eine Rolle spielen. Für einen erfolgreichen Einsatz in der Praxis müssen KI-Systeme menschzentriert gestaltet sein. „Menschzentrierte KI“ beschreibt einen Ansatz, bei dem KI-Systeme nicht als isolierte Technologie, sondern als Bestandteil eines größeren, soziotechnischen Systems gesehen werden [8]. Sie stellt daher den Menschen in den Mittelpunkt und orientiert sich an dessen Bedürfnisses, Werten und Nutzungsanforderungen. Die Interaktion mit dem System sollte für die Nutzer:innen möglichst intuitiv und verständlich sein, um die Akzeptanz zu steigern.

2 Faktoren, die den Umgang mit KI-Systemen maßgeblich beeinflussen, sind die mentalen Modelle der Anwender:innen und ihr Vertrauen in die Technologie. Mentale Modelle [9] beziehen sich hierbei auf die inneren Vorstellungen, die Personen über ein Objekt, zum Beispiel eine KI-Anwendung, haben. Sie bestimmen, wie gut Nutzer:innen ein System verstehen und wie sicher sie sich im Umgang damit fühlen. Vertrauen wiederum ist ein Schlüsselfaktor, der beeinflusst, ob und wie ein System genutzt wird [10]. Beide Aspekte – mentale Modelle und Vertrauen – unterscheiden sich von Person zu Person und hängen stark von deren jeweiliger Rolle im medizinischen Kontext ab.

In der Medizin treffen unterschiedliche Nutzergruppen mit individuellen Anforderungen, Erwartungen und Expertise aufeinander: Patient:innen und ihre Angehörigen, Ärzt:innen, Pflegepersonal sowie Verwaltungsmitarbeiter:innen in Kliniken. Jede dieser exemplarisch genannten Gruppen bringt eigene mentale Modelle und Erfahrungen im Umgang mit Technologie im Allgemeinen und KI im Spezifischen mit. Ein gemeinsames Verständnis über die Funktionsweise von KI, insbesondere in Diagnose- und Behandlungskontexten, ist essenziell, um Missverständnisse und Fehlinterpretationen zu vermeiden.

Betrachten wir beispielhaft die Anforderungen verschiedener Nutzergruppen an ein KI-System zur Diagnoseunterstützung, das Bilddaten auf Erkrankungen überprüft:

- *Die Ärztin oder der Arzt* könnte das System vor allem bei unklaren Befunden einsetzen, um eine Zweitmeinung einzuholen. Für sie/ihn ist wichtig, dass die Ergebnisse zuverlässig und nachvollziehbar sind, da dies nicht nur die Qualität der Diagnose erhöht, sondern auch wertvolle Zeit spart.
- *Das Krankenhausmanagement* hingegen könnte den flächendeckenden Einsatz des Systems in allen Diagnoseprozessen bevorzugen, um statistisch vergleichbare Daten über Diagnosen und deren Übereinstimmung mit den ärztlichen Bewertungen zu erhalten.
- *Für Patient:innen und Angehörige* liegt der Fokus oft darauf, dass die KI-gestützte Diagnose gut verständlich erklärt wird und sie sich von den behandelnden Ärzt:innen umfassend betreut fühlen.

Dieses Beispiel zeigt, dass Entwickler von KI-Systemen nicht nur die technische Umsetzung, sondern auch die Bedürfnisse und Erwartungen der Nutzergruppen berücksichtigen müssen. Gleichzeitig sollten Kliniken und andere Anwender:innen im Gesundheitswesen sorgfältig planen, wie KI-Systeme in bestehende Prozesse integriert werden können. Nur wenn die Perspektiven aller Beteiligten berücksichtigt werden, kann das Potenzial von KI-Anwendungen voll ausgeschöpft werden.

## Von Qualitätskriterien zu Prüfkriterien

**Abb. 2:**
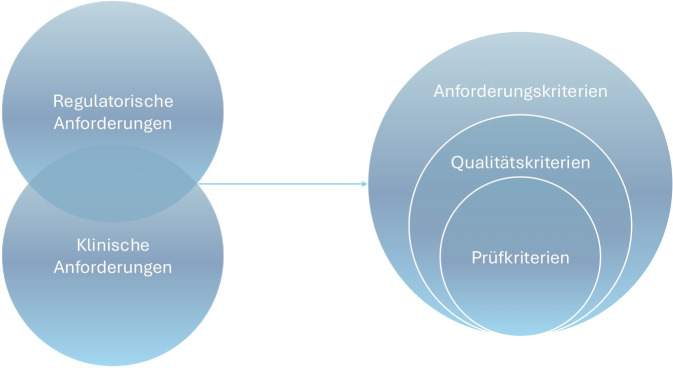
Vom Anforderungsprofil über Qualitätskriterien zu Prüfkriterien

Im vorherigen Kapitel wurden einige regulatorische, klinische und nutzerzentrierte Anforderungen an KI im medizinischen Kontext dargestellt. Sowohl in den klinisch-fachlichen [5] als auch in den regulatorischen Anforderungen (z. B. EU AI Act) finden sich zentrale Kriterien wie „Robustheit“, „Transparenz“, „Genauigkeit“, „Erklärbarkeit“, „Fairness“, „Nutzbarkeit“ u. v. w., die sich auf die KI als technisches Tool beziehen. Diese Kriterien müssen ggf. in Qualitätskriterien für KI umformuliert werden, ohne Inhalt und Bedeutung zu verlieren. Bei der Prüfung von KI-Systemen, deren Ziel es ist, die anforderungsgerechte Aufgabenerfüllung der KI zu bestätigen, sollte die „Prüfbarkeit“ bei der Definition von Qualitätskriterien berücksichtigt werden, damit diese auch als konkrete Prüfkriterien eingesetzt werden können (Abb. 2). Prüfkriterien sind insbesondere jene Qualitätskriterien, für die geeignete Prüfwerkzeuge existieren, mit denen eine KI gezielt auf das jeweilige Kriterium überprüft werden kann. Das spielt auch in der forensischen Analyse von KI-Methoden eine wichtige Rolle, also bei der systematischen Untersuchung im Falle einer Fehlprognose.

Prüfwerkzeuge basieren oftmals auf informationstheoretischen Methoden, die von der Art des KI-Modells abhängen, da diese modellspezifische Komponenten beinhalten. Dadurch entstehen 2 große Herausforderungen:

1. Abhängig vom jeweiligen KI-Modell müssen die Prüfmethoden, sofern sie modellspezifisch ausgelegt sind, angepasst werden. Das kann gerade bei häufigen Veränderungen der Modelle oder auch aufgrund der Vielfalt der verschiedenen Modelle zu einem erheblichen Entwicklungsaufwand führen. In solchen Fällen ist sicherzustellen, dass sich die Prüfwerkzeuge konsistent und parallel zur Modellentwicklung weiterentwickeln. Die Bewertung eines Vorgängermodells mithilfe eines Prüfwerkzeugs sollte auch nach der Aktualisierung des Modells (und des entsprechenden Prüfwerkzeugs) vergleichbar sein.
2. Die KI-Modelle sind mit der Zeit immer komplexer geworden, wodurch deren Nachvollziehbarkeit stark gesunken ist. Das betrifft sowohl die technische Prüfbarkeit dieser Modelle als auch die Nachvollziehbarkeit für die Nutzer:innen. Zugleich erhöht sich mit steigender Komplexität der Aufwand für die Entwicklung geeigneter Prüfkriterien und Prüfwerkzeuge.

## Erklärbarkeit als Qualitätskriterium

Ein wichtiges Qualitätskriterium für den Einsatz von KI-Modellen im medizinischen Kontext ist die Erklärbarkeit dieser Modelle. Das Vorhersageverhalten von tiefen neuronalen Netzen ist jedoch intransparent und undurchsichtig, was durch die erhöhte Modellkomplexität noch weiter verstärkt wird. Zwar weisen Forschende wie London [11] darauf hin, dass Entscheidungen in der Medizin – auch ohne Einsatz von KI-Systemen – nicht immer inhärent transparent sind, doch der Einsatz von nicht nachvollziehbaren KI-Systemen ist weder rechtlich umsetzbar noch ethisch wünschenswert. Daher besteht die Notwendigkeit beim Einsatz von KI-Systemen, die Reliabilität ihrer Funktionsweise und Ergebnisse zu überprüfen und Nutzer:innen die Möglichkeit zu geben, diese nachzuvollziehen. Diese Herausforderung wird in den letzten Jahren insbesondere im Forschungsgebiet der „erklärbaren künstlichen Intelligenz“ (Explainable Artificial Intelligence, XAI) aufgegriffen.

**Abb. 3:**
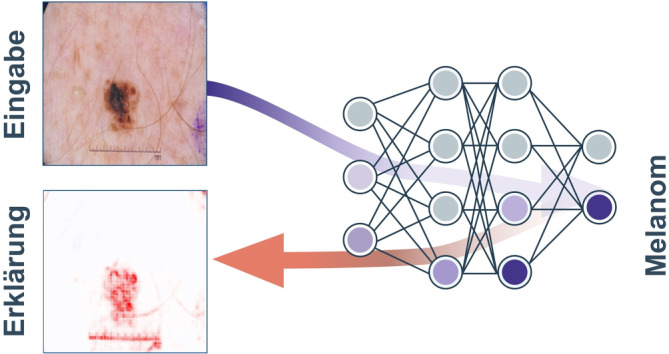
Lokale Erklärung mittels Relevanzkarte für die Vorhersage eines bildbasierten KI-Modells zur Hautkrebserkennung. *Rot* hervorgehoben sind die Eingabewerte, die einen Einfluss auf die KI-Vorhersage haben. Zusätzlich zum Hautmerkmal hat die KI die Messskala (Störfaktor) fokussiert

Traditionelle *lokale Erklärbarkeitsmethoden*, also Erklärmethoden, die darauf abzielen, einzelne KI-Vorhersagen nachvollziehbar zur machen, beispielsweise „Layer-wise Relevance Propagation“ (LRP; [12]) oder GradCAM [13], berechnen sogenannte Relevanzwerte für die jeweiligen Eingabewerte. Dabei kann es sich um Pixelwerte im Fall der bildbasierten Klassifizierung handeln oder um Token (bzw. Wörter) für Sprachmodelle. Die für das Modellverhalten berechneten Relevanzwerte werden häufig in Form von Relevanzkarten visualisiert (Beispiel in Abb. 3). Diese können von Fachleuten wie Ärzt:innen ausgewertet werden, um sicherzustellen, dass die vom Modell verwendeten Eingabewerte mit den Erwartungen der Expert:innen übereinstimmen. Dies ist von Bedeutung, da sich Vorhersagen von KI-Modellen nicht zwingend auf die aus fachlicher Sicht relevanten Merkmale stützen, sondern dazu neigen können, auf Basis irreführender Korrelationen sogenannte Abkürzungen zu entwickeln. Dieses Phänomen ist auch als „Clever-Hans-Effekt“ bekannt [14] und hat seinen Ursprung in der Verhaltensbiologie [15, 16]. Strukturierte Analysen lokaler Erklärungen können dabei unterstützen, Fehler frühzeitig zu erkennen und zu beheben. In Abb. 3 wird am Beispiel eines bildbasierten KI-Modells zur Hautkrebserkennung eine lokale Erklärung in Form einer Relevanzkarte gezeigt. Es ist erkennbar, dass das Modell neben dem Hautmerkmal auch Informationen von Störfaktoren, z. B. der Messskala, nutzt.

In den letzten Jahren hat sich der Fokus in der Erklärbarkeitsforschung zunehmend von lokalen Erklärungen hin zu *globalen Erklärungen* verschoben. Diese zielen darauf ab, die allgemeinen Repräsentationsmechanismen von KI-Modellen zu verstehen. Dabei geht es insbesondere um die Frage, welche Konzepte intern im Modell kodiert sind und welche ggf. nicht. Das Ziel besteht darin, das KI-Modell zu dekodieren, um Substrukturen zu identifizieren, die mit für Menschen verständlichen Konzepten übereinstimmen. Bei diesen Substrukturen handelt es sich beispielsweise um einzelne Neuronen eines neuronalen Netzwerks, wie bei den Erklärbarkeitsmethoden „Network Dissection“ [17] und „Concept Relevance Propagation“ [18], oder lineare Richtungen im latenten Aktivierungsraum des KI-Modells. Konzeptaktivierungsvektoren (Concept Activation Vectors, CAVs; [19]) haben sich als beliebter globaler Erklärbarkeitsansatz etabliert, um zu überprüfen, wie sensibel ein KI-Modell auf erwartete Konzepte für Modellvorhersagen reagiert. Aktuelle Arbeiten wie der „SemanticLens“-Ansatz [20] gehen noch einen Schritt weiter und erlauben eine systematische Erklärung und Validierung aller internen Entscheidungsprozesse in einem KI-Modell. Damit lässt sich nicht nur nachvollziehen, welche Konzepte das Modell intern repräsentieren kann, sondern auch Schritt für Schritt (d. h. Modellebene für Modellebene) erklären, wie das Modell zu seiner Entscheidung kommt, an welcher Stelle und wie stark einzelne Konzepte zur Entscheidung beitragen und aus welchen Trainingsbeispielen diese Konzepte gelernt wurden. Dieser neue, holistische Ansatz des „Erklärens“ erlaubt es, das Modellverhalten gegenüber vorgegebenen Richtlinien oder Regeln zu validieren.

Zum Beispiel kann für bildbasierte KI-Modelle zur Hautkrebserkennung geprüft werden, ob sich das Modell an die „ABCDE“-Regeln zur Erkennung von Melanomen hält und beispielsweise das Konzept „irreguläre Ränder“ (B-Regel bzw. Border-Regel) repräsentiert und es bei der Erkennung von Melanomen auch aktiv nutzt. Dies ist nützlich, um globale Aussagen über von Menschen interpretierbare Konzepte zu treffen, die vom Modell verwendet werden, anstatt Schlussfolgerungen aus der Analyse von Relevanzkarten für einzelne Modellvorhersagen zu ziehen. Da die Genauigkeit der Konzeptrichtung eine zentrale Rolle spielt, ist deren Präzisierung zur saubereren Konzeptdarstellung, beispielsweise durch iterative Konzeptannotation in den Trainingsdaten, ein aktives Forschungsthema [21].

**Abb. 4:**
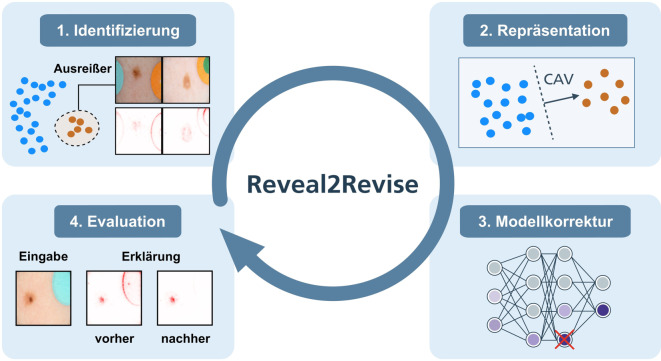
4 Schritte im Framework „Reveal2Revise“ zur iterativen Identifizierung und Korrektur von KI-Fehlverhalten. (*CAV* Concept Activation Vectors)

Über das einfache Verständnis und die Validierung des Modellverhaltens hinaus können Erklärungen auch genutzt werden, um KI-Modelle zu korrigieren. Insbesondere können unerwünschte Konzepte, die beispielsweise durch irreführende Korrelationen verursacht werden, gezielt verlernt werden, um das Modell mit menschlichen Erwartungen in Einklang zu bringen. Während der Entwicklung und im Einsatz von KI-Modellen können diese kontinuierlich auf die Gültigkeit von genutzten Konzepten überprüft werden und verdächtiges Fehlverhalten, welches beispielsweise durch vom Modell aufgegriffene Störfaktoren in den Trainingsdaten verursacht wird, kann markiert werden. Nach der Modellkorrektur kann die Sensitivität des Modells gegenüber dem Störfaktor erneut bewertet werden. Dieser Prozess ist beispielsweise im Framework zum „Erklärenden Interaktiven Maschinellen Lernen“ [22, 23] und im „Reveal2Revise-Framework“ [24] skizziert. Letzteres wird in Abb. 4 dargestellt und umfasst die folgenden Schritte:

1. *Identifizierung*: Einsatz globaler Erklärbarkeitsmethoden zur Identifizierung von Fehlverhalten des Modells;
2. *Repräsentation des Störfaktors:* Identifizierung der Modellsubstruktur, die das Fehlverhalten kodiert;
3. *Modellkorrektur*: Modellkorrektur, um das Fehlverhalten gezielt zu verlernen;
4. *Evaluation*: Bewertung, ob das Fehlverhalten erfolgreich verlernt wurde und ob das Modell weiterhin gut funktioniert.

## Ein Prüfschema für KI in der Medizin

Im Folgenden stellen wir ein Prüfschema für KI vor, das beispielsweise im „Projekt 540“ eingesetzt wurde, das im Auftrag des Bundesamts für Sicherheit in der Informationstechnik (BSI) durchgeführt wurde [25]. Das Prüfschema bewertet KI-Systeme auf Basis von Prüfkriterien, die jene Qualitätskriterien widerspiegeln, welche aus den Anforderungen abgeleitet und als prüfbar eingestuft wurden. Für jedes Prüfkriterium sind geeignete Metriken festzulegen, die in den jeweiligen Prüfmethoden verwendet werden, um das Modell systematisch zu bewerten.

In [25] wurden bekannte Prüfkriterien und Prüfmethoden für medizinische Diagnosesysteme exemplarisch anhand der Analyse von Elektrokardiogrammen vorgestellt und deren Einsatzfähigkeit analysiert. Je nach Anwendungsfall und Zweck der Prüfung können die Prüfkriterien und Prüfmethoden in Umfang und Durchführung variieren (die in [25] genutzten Prüfkriterien und Prüfmethoden sind in Tab. 1 dargestellt). Ein einzelnes Prüfkriterium kann dabei unter verschiedenen Bedingungen geprüft bzw. gemessen werden, wodurch eine Reihe von Messergebnissen für das Modell entsteht. Solche Bedingungen können beispielsweise verschiedene Subklassen der Daten oder schwer vorhersehbare Sonderfälle umfassen.

**Tab. 1:** Prüfkriterien und Prüfmethoden für KI-Systeme im „Projekt 540“ des Bundesamts für Sicherheit in der Informationstechnik (BSI; [25])

Prüfkriterium	Prüfmethoden
*Leistungsfähigkeit*	Messung der Modellleistung mittels geeigneter Metriken unter normalen Bedingungen (d. h. auf unveränderten Daten)
*Robustheit*	Messung der Modellleistung mittels geeigneter Metriken unter Bedingungen mit Störeinflüssen (d. h. auf gestörten Daten)
*Unsicherheit*	Messen von Wahrscheinlichkeiten und Standardabweichungen von mehreren Simulationen des Modells (z. B. „Monte-Carlo Dropout“ [26])
*Erklärbarkeit*	Erklärung von Modellvorhersagen mittels geeigneter Erklärbarkeitsmethoden (z. B. TCAV [19])

*TCAV* Testing with Concept Activation Vectors

**Abb. 5:**
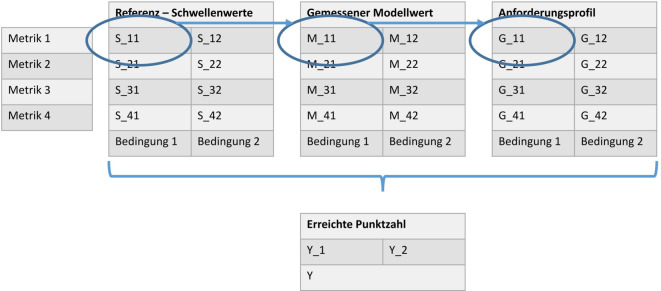
Visualisierung eines Prüfschemas. In der Tabelle „Referenz – Schwellenwerte“ sind die Werte hinterlegt, die ein Modell unter verschiedenen Bedingungen je Metrik erreichen muss, um die Prüfung bestanden zu haben. In der Tabelle „Anforderungsprofil“ können Gewichte hinterlegt werden, um eine Gewichtung der Prüfkriterien vorzunehmen. Diese Gewichte stellen die erreichten Punkte dar, sollte der gemessene Modellwert (Tabelle „Gemessener Modellwert“) den Referenzwert überschritten haben. Am Ende werden die Punkte entsprechend über alle Metriken und Bedingungen aufsummiert, um eine Gesamtpunktzahl zu erhalten

Die Messergebnisse müssen mit Referenzwerten verglichen werden (Abb. 5), anhand derer sich die Qualität des Modells beurteilen lässt. Solche Referenzwerte können beispielsweise ein Goldstandard oder von Expertengremien festgelegte Zielwerte sein. Die Messwerte und die Referenzwerte werden einander gegenübergestellt und auf Grundlage eines zuvor definierten Punktesystems bewertet, das als Anforderungsprofil dient. Dieses Punktesystem kann auch individuell angepasst werden, sodass die fachspezifischen Bedürfnisse der Nutzer:innen entsprechend gewichtet berücksichtigt werden können. Die erzielten Punkte werden zusammengerechnet und ergeben den erreichten Punktewert. So lassen sich dann auch quantitative Qualitätsbewertungen erstellen.

## Standardisierung von KI-Qualitätskriterien in der Medizin

Die Entwicklung von neuen Standards spielt eine wichtige Rolle in der sicheren Anwendung von KI in der Medizin. Sie stellen nicht nur interoperable Schnittstellen sicher oder decken wesentliche Aspekte der Cybersicherheit ab, sondern befassen sich auch direkt mit Aspekten der Prüfung und Zertifizierung von KI-Systemen, da sich diese von bisheriger Software unterscheiden.

Standards werden in Gremien und Arbeitsgruppen verschiedener Standardisierungsorganisationen entwickelt – auf nationaler Ebene beim Deutschen Institut für Normung (DIN) und der Deutschen Kommission Elektrotechnik Elektronik Informationstechnik (DKE), auf europäischer Ebene beim Europäischen Komitee für Normung (CEN), dem Europäischen Komitee für elektrotechnische Normung (CENELEC) und dem Europäischen Institut für Telekommunikationsnormen (ETSI) sowie auf internationaler Ebene bei der Internationalen Fernmeldeunion (ITU) und der Internationalen Organisation für Normung (ISO). Ein besonderer Fokus der aktuellen Entwicklungen von KI-Standards liegt dabei auf deren Harmonisierung, z. B. mit dem aktuellen Forschungsstand oder im länderübergreifenden Kontext. Hinsichtlich KI gibt es zahlreiche Standards, die auf internationaler Ebene entwickelt worden sind, sowie wegweisende Strategiepapiere, die die nationalen Interessen widerspiegeln. In Deutschland wurde im Auftrag des damaligen Bundesministeriums für Wirtschaft und Klimaschutz die „Deutsche Normungsroadmap Künstliche Intelligenz“ [27] im Rahmen der KI-Bundesstrategie verfasst. Über 570 Expert:innen aus verschiedenen Bereichen, u. a. der Forschung, der Unternehmen und der öffentlichen Hand, haben zu 9 verschiedenen Schwerpunktthemen den aktuellen Status quo erarbeitet sowie Handlungsbedarfe identifiziert. Eins der 9 Schwerpunktthemen ist dabei die Medizin. Aus der Normungsroadmap entstanden zahlreiche Umsetzungsprojekte, u. a. die Entwicklung der DIN SPEC 92006 „Künstliche Intelligenz – Anforderungen an KI-Prüfwerkzeuge“.

Auch auf internationaler Ebene ist die Erarbeitung von Standards für KI in der Medizin ein aktives Ziel. Die internationale Standardisierungsinitiative der Organisationen der Vereinten Nationen (UN) – der ITU, der WHO und der Weltorganisation für geistiges Eigentum (WIPO) – mit dem Titel „Global Initiative AI for Health“ ([28]; zuvor ITU/WHO „Focus Group AI for Health“ [6, 29]) entwickelt in multidisziplinärer Zusammenarbeit Best Practices zu Fragen der Governance, Softwareentwicklung sowie zu Benchmarking-Prozessen für verschiedene medizinische KI-Anwendungsfälle, wie beispielsweise die KI-gestützte digitale Mikroskopie zur Erkennung von Gebärmutterhalskrebs [30]. Seit 2018 sind bereits über 2000 Seiten an Best Practices für ethische, regulatorische, technische und klinische Anforderungen entwickelt und von der ITU und WHO veröffentlicht worden (Beispiele siehe [7, 31, 32]). Zusätzlich zu der Erarbeitung und Weiterentwicklung von KI-Standards sowie Prüfmethoden und -werkzeugen für KI steht auch die praktische Umsetzung und Anwendung dieser Best Practices für diverse KI-Systeme zur lokalen und nachhaltigen Implementierung im Fokus der Gruppe. Besonders berücksichtigt werden hierbei Länder mit niedrigem und mittlerem Einkommen.

## Fazit

Es steht außer Frage, dass der Einsatz von KI in der Medizin erhebliches Potenzial für Fortschritte bietet, welches jedoch noch nicht vollständig ausgeschöpft ist. Ebenso klar ist es, dass wir KI in der Medizin nicht bedingungslos vertrauen können. Um Vertrauen in die Technologie zu schaffen, müssen verschiedene Perspektiven – darunter die der Entwickler:innen, Anwender:innen und Patient:innen – berücksichtigt werden. Daher ist es notwendig, ein multidisziplinäres Vorgehen zur Entwicklung angemessener Qualitätskriterien und Prüfwerkzeuge zu verfolgen. Die Erklärbarkeit von KI-Modellen und die Standardisierung von Prüfverfahren sind dabei wesentliche Bestandteile. Die Umsetzung sollte eine menschzentrierte Perspektive einnehmen, die Zuverlässigkeit von Tests, Sicherheit und Vertrauenswürdigkeit in den Fokus setzt (siehe z. B. [33]). Das Nutzen-Risiko-Verhältnis sowie andere diskutierte Anforderungen, Qualitätskriterien und Prüfkriterien dieses Artikels können je nach medizinischem Anwendungsfall variieren.

Abschließend lässt sich feststellen, dass bereits gute Lösungsansätze existieren. Um diese jedoch erfolgreich in der Praxis zu implementieren, sind kontinuierliche Forschungsanstrengungen erforderlich, insbesondere angesichts der dynamischen Entwicklungen im technologischen Bereich.

## References

[1] Bundesministerium der Justiz Medizinprodukterecht-Durchführungsgesetz (MPDG). https://www.gesetze-im-internet.de/mpdg/. Zugegriffen: 14. Apr. 2025

[2] European Parliament and Council of the European Union. Medical Device Regulation (2017on) Regulation (EU) 2017/745 of the European Parliament and of the Council of 5 April 2017 on medical devices. https://data.europa.eu/eli/reg/2017/745/2025-01-10. Zugegriffen: 14. Apr. 2025

[3] Regulation (EU) 2024/1689 of the European Parliament and of the Council of 13 June 2024 laying down harmonised rules on artificial intelligence and amending Regulations (EC) No 300/2008, (EU) No 167/2013, (EU) No 168/2013, (EU) 2018/858, (EU) 2018/1139 and (EU) 2019/2144 and Directives 2014/90/EU, (EU) 2016/797 and (EU) 2020/1828 (Artificial Intelligence Act) (Text with EEA relevance). PE/24/2024/REV/1, OJ L, 2024/1689, 12.07.2024, ELI: http://data.europa.eu/eli/reg/2024/1689/oj (BG, ES, CS, DA, DE, ET, EL, EN, FR, GA, HR, IT, LV, LT, HU, MT, NL, PL, PT, RO, SK, SL, FI, SV). http://data.europa.eu/eli/reg/2024/1689/oj. Zugegriffen: 14. April 2025

[4] Beede E, Baylor E, Hersch F et al (2020) A human-centered evaluation of a deep learning system deployed in clinics for the detection of diabetic retinopathy. In: Proceedings of the 2020 CHI conference on human factors in computing systems, S 1–12 10.1145/3313831.3376718

[5] International Telecommunication Union (ITU) (2023) FG-AI4H DEL7.4—Clinical evaluation of AI for health. https://www.itu.int/dms_pub/itu-t/opb/fg/T-FG-AI4H-2023-3-PDF-E.pdf. Zugegriffen: 14. Apr. 2025

[6] International Telecommunication Union Focus group on AI for health. https://www.itu.int/en/ITU-T/focusgroups/ai4h/Pages/default.aspx. Zugegriffen: 14. Apr. 2025

[7] World Health Organization (2021) Ethics and governance of artificial intelligence for health. https://iris.who.int/bitstream/handle/10665/341996/9789240029200-eng.pdf?sequence=1. Zugegriffen: 14. Apr. 2025

[8] Riedl MO (2019) Human-centered artificial intelligence and machine learning. Hum Behav Emerg Technol 1(1):33–36. 10.48550/arXiv.1901.11184

[9] Johnson-Laird PN (1983) Mental models: towards a cognitive science of language, inference, and consciousness. Harvard University Press. ISBN 978-0-674-56882‑2.

[10] Lee JD, See KA (2004) Trust in automation: designing for appropriate reliance. Hum Factors 46(1):50–80. 10.1518/hfes.46.1.50.30392 15151155 10.1518/hfes.46.1.50_30392

[11] London AJ (2019) Artificial intelligence and black-box medical decisions: accuracy versus explainability. Hastings Cent Rep 49(1):15–21. 10.1002/hast.973 30790315 10.1002/hast.973

[12] Bach S, Binder A, Montavon G, Klauschen F, Müller KR, Samek W (2015) On pixel-wise explanations for non-linear classifier decisions by layer-wise relevance propagation. PLoS ONE 10(7):e130140. 10.1371/journal.pone.0130140 26161953 10.1371/journal.pone.0130140 PMC4498753

[13] Selvaraju RR, Cogswell M, Das A, Vedantam R, Parikh D, Batra D (2020) Grad-CAM: visual explanations from deep networks via gradient-based localization. Int J Comput Vis 128:336–359

[14] Lapuschkin S, Wäldchen S, Binder A, Montavon G, Samek W, Müller KR (2019) Unmasking Clever Hans predictors and assessing what machines really learn. Nat Commun 10(1):1096. 10.1038/s41467-019-08987-4 30858366 10.1038/s41467-019-08987-4 PMC6411769

[15] Pfungst O (1911) Clever Hans:(the horse of Mr. Von Osten.) a contribution to experimental animal and human psychology. Holt, Rinehart and Winston 10.5962/bhl.title.56164

[16] Naguib M, Krause ET (2006) Methoden der Verhaltensbiologie. Springer, Berlin Heidelberg 10.1007/3-540-33495-5

[17] Bau D, Zhou B, Khosla A, Oliva A, Torralba A (2017) Network dissection: quantifying interpretability of deep visual representations. In: Proceedings of the IEEE conference on computer vision and pattern recognition, S 6541–6549 10.1109/cvpr.2017.354

[18] Achtibat R, Dreyer M, Eisenbraun I, Bosse S, Wiegand T, Samek W, Lapuschkin S (2023) From attribution maps to human-understandable explanations through concept relevance propagation. Nat Mach Intell 5(9):1006–1019. 10.1038/s42256-023-00711-8

[19] Kim B, Wattenberg M, Gilmer J, Cai C, Wexler J, Viegas F (2018) Interpretability beyond feature attribution: quantitative testing with concept activation vectors (tcav). In: International conference on machine learning PMLR, S 2668–2677 10.48550/arxiv.1711.11279

[20] Dreyer M, Berend J, Labarta T et al (2025) Mechanistic understanding and validation of large AI models with SemanticLens. arXiv preprint arXiv:2501.05398. 10.48550/arXiv.2501.05398

[21] Pahde F, Wiegand T, Lapuschkin S, Samek W (2025) Ensuring medical AI safety: explainable AI-driven detection and mitigation of spurious model behavior and associated data. arXiv preprint arXiv:2501.13818. 10.48550/arXiv.2501.13818 10.1007/s10994-025-06834-w PMC12343733 40814399

[22] Teso S, Kersting K (2019) Explanatory interactive machine learning. In: Proceedings of the 2019 AAAI/ACM Conference on AI, Ethics, and Society, S 239–245 10.1145/3306618.3314293

[23] Schramowski P, Stammer W, Teso S et al (2020) Making deep neural networks right for the right scientific reasons by interacting with their explanations. Nat Mach Intell 2(8):476–486. 10.1038/s42256-020-0212-3

[24] Pahde F, Dreyer M, Samek W, Lapuschkin S (2023) Reveal to revise: An explainable ai life cycle for iterative bias correction of deep models. In: International Conference on Medical Image Computing and Computer-Assisted Intervention. Springer, Cham, S 596–606 10.1007/978-3-031-43895-0_56

[25] Bundesamt für Sicherheit in der Informationstechnik Projekt P540: Ergebnisse und Empfehlungen: „Einsatz von Künstlicher Intelligenz in medizinischen Diagnose- und Prognosesystemen“. Abgerufen am [30.01.2025]. https://www.bsi.bund.de/SharedDocs/Downloads/DE/BSI/Publikationen/Studien/Projekt_P540/Projekt_P540.pdf?__blob=publicationFile&v=4. Zugegriffen: 14. Apr. 2025

[26] Gal Y, Ghahramani Z (2016) Dropout as a Bayesian approximation: representing model uncertainty in deep learning. In: Proceedings of the 33rd International Conference on International Conference on Machine Learning ICML’16, New York. Bd. 48, S 1050–1059

[27] DIN, DKE (2022) Deutsche Normungsroadmap Künstliche Intelligenz (Ausgabe 2). www.din.de/go/normungsroadmapki. Zugegriffen: 14. Apr. 2025

[28] World Health Organization Global initiative on AI for health. https://www.who.int/initiatives/global-initiative-on-ai-for-health. Zugegriffen: 14. Apr. 2025

[29] Wiegand T, Krishnamurthy R, Kuglitsch M et al (2019) WHO and ITU establish benchmarking process for artificial intelligence in health. Lancet 394(10192):9–11. 10.1016/S0140-6736(19)30762-7 30935732 10.1016/S0140-6736(19)30762-7

[30] Holmström O, Linder N, Kaingu H et al (2021) Point-of-care digital cytology with artificial intelligence for cervical cancer screening in a resource-limited setting. JAMA Netw Open 4(3):e211740. 10.1001/jamanetworkopen.2021.1740 33729503 10.1001/jamanetworkopen.2021.1740 PMC7970338

[31] World Health Organization (2023) Regulatory considerations on artificial intelligence for health. https://iris.who.int/bitstream/handle/10665/373421/9789240078871-eng.pdf. Zugegriffen: 14. Apr. 2025

[32] International Telecommunication Union Focus group on AI for health: deliverables. https://www.itu.int/en/ITU-T/focusgroups/ai4h/Pages/deliverables.aspx. Zugegriffen: 14. Apr. 2025

[33] Shneiderman B (2020) Human-centered artificial intelligence: reliable, safe & trustworthy. Int J Human Comput Interact 36(6):495–504. 10.1080/10447318.2020.1741118

